# Systematic Review of Potential Anticancerous Activities of *Erythrina senegalensis DC (Fabaceae)*

**DOI:** 10.3390/plants11010019

**Published:** 2021-12-22

**Authors:** Souleymane Fofana, Moussa Ouédraogo, Rafaèle Calvo Esposito, Windbedema Prisca Ouedraogo, Cédric Delporte, Pierre Van Antwerpen, Véronique Mathieu, Innocent Pierre Guissou

**Affiliations:** 1 Laboratory of Drug Science, Higher Institute of Health Sciences (INSSA), Nazi BONI University, Bobo-Dioulasso P.O. Box 1091, Burkina Faso; fof_soul@yahoo.fr; 2 Laboratory of Drug Development (LADME), Training and Research Unit, Health Sciences, Joseph KI-ZERBO University, Ouagadougou P.O. Box 7021, Burkina Faso; Ouemoussa10@gmail.com (M.O.); windbedma@gmail.com (W.P.O.); 3 Department of Pharmacotherapy and Pharmaceuticals, Faculty of Pharmacy, Université Libre de Bruxelles (ULB), 1050 Brussels, Belgium; Rafaele.Calvo.Esposito@ulb.be; 4 Protein Chemistry Unit, Department of General Chemistry I, Faculty of Medicine, Université Libre de Bruxelles, 1070 Brussels, Belgium; 5 RD3-Pharmacognosy, Bioanalysis and Drug Discovery Unit and Analytical Platform, Faculty of Pharmacy, Universite’ Libre de Bruxelles (ULB), 1050 Brussels, Belgium; Cedric.Delporte@ulb.be (C.D.); pierre.van.antwerpen@ulb.be (P.V.A.); 6 ULB Cancer Research Center, Université Libre de Bruxelles (ULB), 1050 Bruxelles, Belgium; 7 Faculty of Health Sciences, Saint Thomas d’Aquin University, Ouagadougou P.O. Box 10212, Burkina Faso

**Keywords:** *Erythrina senegalensis*, prenylated isoflavonoid, erysenegalensein, alpinumisoflavone, anticancer

## Abstract

The objective of this study was to carry out a systematic review of the substances isolated from the African medicinal plant *Erythrina senegalensis,* focusing on compounds harboring activities against cancer models detailed in depth herein at both in vitro and in vivo preclinical levels. The review was conducted through Pubmed and Google Scholar. Nineteen out of the forty-two secondary metabolites isolated to date from *E. senegalensis* displayed interesting in vitro and/or in vivo antitumor activities. They belonged to alkaloid (Erysodine), triterpenes (Erythrodiol, maniladiol, oleanolic acid), prenylated isoflavonoids (senegalensin, erysenegalensein E, erysenegalensein M, alpinumisoflavone, derrone, warangalone), flavonoids (erythrisenegalone, senegalensein, lupinifolin, carpachromene) and pterocarpans (erybraedine A, erybraedine C, phaseollin). Among the isoflavonoids called “erysenegalensein”, only erysenealenseins E and M have been tested for their anticancerous properties and turned out to be cytotoxic. Although the stem bark is the most frequently used part of the plant, all pterocarpans were isolated from roots and all alkaloids from seeds. The mechanisms of action of its metabolites include apoptosis, pyroptosis, autophagy and mitophagy via the modulation of cytoplasmic proteins, miRNA and enzymes involved in critical pathways deregulated in cancer. Alpinumisoflavone and oleanolic acid were studied in a broad spectrum of cancer models both in vitro and in preclinical models in vivo with promising results. Other metabolites, including carpachromen, phaseollin, erybraedin A, erysenegalensein M and maniladiol need to be further investigated, as they display potent in vitro effects.

## 1. Introduction

Cancer is a leading cause of death worldwide, accounting for nearly 10 million deaths in 2020, 70% of which occurred in low- and middle-income countries due to late-stage discoveries, diagnosis inability and poor access to appropriate treatment [1]. The plant kingdom has always been an inexhaustible source of medicine and food for man. More than 3000 plants worldwide have been reported to have anticancer properties [2]. In particular, the plant kingdom has been the source or the inspiration for 208 anticancer drugs (41%) that have been marketed from 1981 to 2019 [3]. In the USA 40% of the approved molecules are natural compounds or inspired by them, from which, 74% are used as anti-cancer drugs [4]. Therefore, despite the rise in the development of targeted therapies against cancer, Nature is regaining interest in therapeutics in general and, in particular, those that can be used against cancer. Many substances of plant origin have been shown to display significant anticancer potential by targeting proteins that are specifically deregulated in cancer [4,5].

*Erythrina* is a genus of dicotyledonous plants belonging to the Fabaceae family, which includes 129 species distributed in the tropic and subtropic regions [6]. Several species of Erythrina harbor large red flowers, from which their name originates (in Greek ερυθρος eruthros means red). *Erythrina senegalensis* is a thorny tree native to West Africa and Sudan where it is one of the oldest used herbals. All parts of the plant i.e., stem bark, roots, leaves, flowers and seeds are used in traditional medicine. Stem bark and root extracts are used in the treatment of asthenia, pain, inflammation, hematuria, rheumatism, jaundice, malaria, dysentery, bronchial infections and many venereal diseases [7,8]. They are also recommended in threatened abortion, female infertility, fibroids, amenorrhea and vomiting. The leaves’ and flowers’ extracts are indicated in the treatment of oral diseases (gingivitis, stomatitis), hematemesis, hemorrhoids and dermatoses [7,8,9]. Due to the fact that cancer remains not considered per se as a disease by African traditional healers, who treat symptoms rather than diseases, it is unclear whether *E. senegalensis* extracts are actually used for this purpose in traditional practice. However, the extracts are used in the traditional treatment of pain, inflammation, asthenia, hematuria and fibroids, which are symptoms that may be associated with several cancer types. 

*Erythrina senegalensis* has been the subject of numerous studies which have led to the identification of phytochemicals with marked biological properties. However, the evaluation of their potential anticancerous properties started much later and is still ongoing. This systematic review aimed to make an inventory of all chemicals having been isolated from *Erythrina senegalensis,* and to further review their potential anticancerous properties described to date. Specifically, we checked for anti-proliferative effects (50% growth inhibitory concentration) on the various cell line models used and make overview of the current knowledge of the mechanisms of action of these compounds. Finally, we highlighted differences between the most promising compounds having already been shown to exert anti-cancer effects in vivo, and in preclinical models in order to discuss their potential in comparison to other natural compounds from other *Erythrina* species. 

## 2. Compounds Isolated from *Erythrina senegalensis*

**Figure 1:**
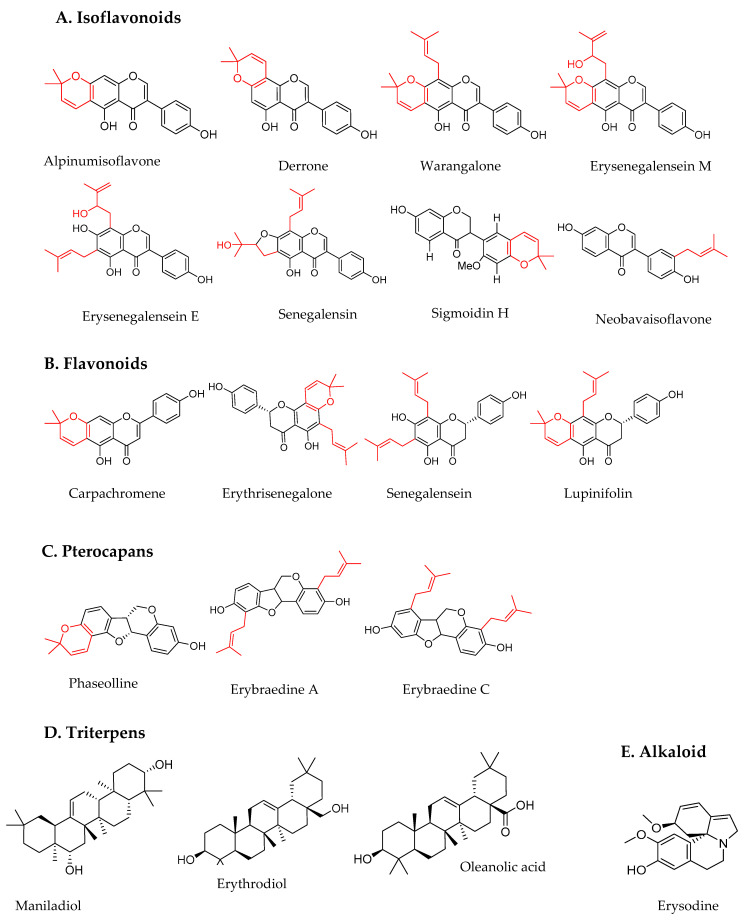
Chemical structures of potential anticancerous compounds isolated from *E. senegalensis.* Flavonoids and isoflavonoids are all prenylated and the prenyl group is highlighted in red. The first 4 isoflavonoids are dimethylpyrano-isoflavonoids.

The phytochemical characterization of *E. senegalensis* started in 1985. Up to date, 31 March 2021, we have recorded only 42 metabolites with elucidated chemical structures isolated from *E. senegalensis* (Table 1, Figure 1 and Supplementary Information Figure S1). These substances are mainly known thanks to the works of a team from Cameroon who have helped to elucidate the chemistry of the majority of the currently known secondary metabolites of this plant. Thirty-two compounds have been isolated from the stem bark of the plant, the most investigated part of the plant to date. Flavonoids counted for 27 of them. One flavone and six pterocarpans, which are isoflavonoid derivatives, have been found exclusively to date in the roots of the plant. It is noteworthy that only three alkaloids and four triterpenes have been isolated from the seeds and the stem bark of *E. senegalensis*, respectively (Table 1, Figure 1, and Supplementary Information Figure S1). All 42 metabolites were extracted using organic solvents, mainly methanol and/or dichloromethane, hexane and chloroform (Table 1). No aqueous or more polar extracts were used to date to isolate compounds from this species.

Among these 42 substances (their structures are detailed in Supplementary Information Figure S1), 19 (45%) have demonstrated potential anticancerous properties (Table 1). They belonged to families of flavonoids, triterpenes (maniladiol, erythrodiol, oleanolic acid), alkaloids (erysodine) and cinnamates (erythrinasinate). Their structures are illustrated in Figure 1. Noteworthy, all these flavonoids, isoflavonoids and pterocarpans are prenylated. To our knowledge, twenty-two compounds, i.e., 52% were not yet tested for their potential anticancerous activity (Table 1).

## 3. In Vitro Anticancerous Activities of Compounds from *E. senegalensis*: Mechanisms of Action

Table 2 details the in vitro effects of each compound on cancer cell line global growth expressed by their 50% inhibitory concentration (IC_50_) on tested cell lines. It is noteworthy that we did not report in this table the results of assays from which the IC_50_ concentration could not be determined. This means that for several products, assays were conducted but the IC_50_ was not reached in the range of concentrations tested. The compounds whose mechanism(s) of action have been investigated deeper are developed below in separated sections.

### 3.1. Isoflavonoids and Flavonoids

Table 2 highlights that carpachromene, a pyrano-flavonoid extracted from the roots of the plant, seems to display the most potent inhibitory effects, with a mean IC_50_ concentration of 23 nM on three cancer cell lines [24]. However, the positive control used in the assay, i.e., 5-FU displayed also a surprisingly very low IC_50_, ranging from 53 to 76 nM (mean GI_50_ according to the NCI of 899 nM on their 60 cancer cell line panel). Experimental conditions may have been different. Further studies should be conducted to confirm the potency found by Sheu et al. in 2005 [24]. In addition, no mechanistic studies have been conducted to date, at least to our best knowledge.

#### 3.1.1. Alpinumisoflavone (AIF)

AIF is an isomer of carpachromene first isolated from the stem bark of *E. senegalensis* by Oh et al. 1999 [16]. Contrary to carpachromene, its cytotoxic effects have been demonstrated and investigated in vitro in many cancer models, with a mean IC_50_ of 35 µM (Table 2). The main cell deaths induced by AIF described to date are apoptosis and pyroptosis, as detailed below.

***AIF induces apoptosis.*** AIF has been shown to trigger apoptosis in various cell models through different mechanisms. In lung cancer cells (H2108 and H1299), AIF induces caspase 3/7 mediated apoptosis by deregulating the ERK/MAPK pathway and the NF-kB pathway [44]. Similarly, an inhibition of STAT3 and NF-κB was also involved in the AIF-induced apoptosis of human promyelocytic leukemia (HL-60, K-562) and lymphoblastic leukemia (MOLT-4) cells [42]. This apoptosis-triggering mechanism appeared different in CCRF-CEM chemosensitive acute lymphoblastic leukemia cells, where a loss of mitochondrial membrane potential, ROS production and the activation of caspases 3/7 were predominant [38]. In colorectal cancer cells (HCT-116, SW480), AIF activates apoptosis by inhibiting the broken double-stranded DNA repair RAD51 pathway [40].

In renal adenocarcinoma (786-O and Caki1) and esophageal squamous cell carcinoma (EC9706 and KYSE30), AIF appears to activate apoptosis, modulating the levels of various miRNAs displaying either oncogenic or tumor suppressor functions [71]. In 786-O and Caki-1 cells, AIF increases the expression of miR-101, which inhibits the Akt pathway via the degradation of its target Ral binding protein 1 (RLIP76) [36]. RLIP76 is a GTPase-activating protein that is required for tumor progression, including cell proliferation, apoptosis evasion and invasion [72]. In EC9706 and KYSE30 cells, it is the miR-370/PIM1 signaling pathway that is activated by AIF [73]. In addition, AIF potentiates the radiosensitivity of these cells, making their DNA more vulnerable to attack by ROS, whose accumulation is caused by the suppression of the antioxidant nuclear transcription factors Nrf2 [45]. AIF thus seems to be able to induce apoptosis through well-known classical pathways, but also through the deregulation of key miRNAs.

***AIF induces pyroptosis and autophagy***. Pyroptosis is a capsase 1-dependent cell death triggered by pro-inflammatory signals that has been observed in hepatocellular carcinoma cells (SMMC 7721, Huh7) following AIF treatment [41]. The classical NLRP3 -caspase-1-IL-1β and IL-18 inflammasome cascade is triggered in addition to autophagic processes, according to the increase in acidic cytoplasmic compartments, beclin-1, LC3II and Atg 5 [41]. Such concomitant autophagy has been shown to be triggered as a protective mechanism against pathogen-induced pyroptosis in macrophages [74]. 

***Other AIF induced effects.*** AIF inhibits the HIF-1α (hypoxia inducible factor alpha) protein in breast cancer T47D cells [39]. This transcription factor acts as a sensor of cellular oxygen, regulating the expression of genes allowing cell survival and stimulating angiogenesis to face hypoxia. Its inhibition therefore leads to a decrease in cell proliferation and angiogenesis within the tumor [75]. AIF also represses properties involved in metastatic processes in various cellular models: it decreases the migration and chemotaxis of MDA-MB-231 metastatic breast cancer cells [39] as well as the adhesion, migration and invasion of melanoma cells [76]. This later effect was attributed to the modulation of COX-2 expression via the miR-124/SphK1 axis [76].

In conclusion, although the active concentration of AIF in vitro may appear quite higher than that of other anticancer agents, the interesting and pleiotropic effects induced by this compound explain the considerable interest it raises to researchers and its investigations in preclinical in vivo models (see below).

#### 3.1.2. Derrone

Derrone is a pyrano-isoflavone isomer of AIF. It was isolated from the trunk bark of *E. senegalensis* by Oh et al. in 1999 [16]. Derrone displays similar in vitro global growth inhibitory effects to AIF in terms of IC_50_ concentrations (24 to 46 µM), except towards the resistant KB-3-1 cell models that appeared even less sensitive to derrone than AIF (Table 2). In A549 lung cancer cells, derrone induces apoptosis and autophagy via an increase in ROS and intracellular calcium concentrations, which is at the origin of the phosphorylation and inhibition of ERKs [49]. Some closed effects can also be observed with respect to AIF in lung cancer and leukemia cells (see above). Nonetheless, its cellular effects are also ascribed to its strong inhibitory effects against Aurora kinases A and B, with the respective IC_50_ of 22.3 µM and 6 µM in direct kinase inhibition assays [50]. Aurora kinases are essential serine/threonine kinase proteins, used during mitosis via their key roles in chromosomal segregation and cell polyploidy [50]. Their overexpression in many cancers including colon, breast, prostate, pancreatic and thyroid cancer is associated with advanced stages and poor prognosis [77]. Their inhibition leads to chromosomal and ploidy aberrations, triggering apoptotic cell death [78].

#### 3.1.3. Warangalone (Scandenone), Auriculatin and Auriculasin

Warangalone is an 8-prenylated AIF isolated by Fomum et al. in 1985 [20] from the stem bark of *E. senegalensis* that appeared more potent than AIF, with a mean IC_50_ below 25 µM (ranging from 7 to 73 µM, this latter value having been observed again in the KB-3-1 resistant model; Table 2). Prenylation has been extensively shown to provide increased in vitro anticancerous activity [79]. The only mechanistic study to date has revealed its ability to trigger caspase 9/3 dependent apoptosis in HL-60 leukemia cells [35].

Auriculatin (2’-hydroxy warangalone) and auriculasin (3’-hydroxy warangalone) are B-ring diol isoflavonoids, which are functional isomers of warangalone. Auriculatin was isolated from *E. senegalensis* by Taylor et al. in 1986 [19], but not auriculasin. To date, only the cytotoxic activity of auriculasin has been studied in prostate cancer lines, where it induces ROS-mediated apoptotic cell death [80], suppresses angiogenesis [81] and sensitizes TRAIL-resistant primary prostate cancer cells to TRAIL-mediated apoptosis via the up-regulation of DR5 and downstream signaling pathways [82].

#### 3.1.4. Erysenegalenseins, Neobavaisoflavone and Sigmoidin H

Erysenegalenseins are isoflavonoids specific to *E. senegalensis* species, at least to date. Among these, only erysenegalensein M and E were investigated for their potential anticancerous activity. Erysenegalensein M is chemically closely related to warangalone, with a hydroxy-prenylated substitution in place of the prenylated substitution on carbone C8 (Figure 1). Erysenegalensein M induces potent in vitro anticancerous effects on LNCaP prostate cancer and MCF-7 breast cancer cell global growth (IC_50_ of 8 µM) similarly to warangalone (IC_50_ of 7 µM), but is inactive on Ishikawa endometrial cancer cells [29]. Regarding erysenegalensein E, the very poor activity towards KB-3-1 cells in comparison to KB cells (10 times less active) may reside in the multidrug resistance phenotype of the KB-3-1 cell line [34]. Whether erysenegalensein E and its analogs are or are not subject to multidrug resistance mechanisms should thus be considered in further studies.

Neobavaisoflavone and sigmoidin H are other isoflavones isolated from the stem bark of *E. senegalensis* by Kuete et al. in 2014 [14]. Neobavaisoflavone is 3′-monoprenylated isoflavonoid, compared to senegalensein and erysenegalenseins (Figure 1), a feature that may explain its lower activity. Nevertheless, authors have compared their in vitro anticancerous properties on chemosensitive models of breast cancer (MDA-MB-231-pcDNA3), colon cancer (HCT116 p53^+/+^), leukemia (CCRF -CEM) and glioblastoma (U87 MG), as well as on corresponding chemoresistant models of breast cancer MDA-MB-231-BCRP-clone 23, colon cancer HCT116 p53^−/−^, CEM/ADR-5000 leukemia and U87MG.ΔEGFR glioblastoma. Due to the fat that the IC_50_ was not reached in several chemosensitive models, they are not reported in Table 2. The ratio of the IC_50_ of metabolites on chemoresistant cancer cell lines/the IC_50_ of the respective metabolites obtained from their corresponding chemosensitive cancer cell lines revealed that the cells were less resistant to neobavaisoflavone or sigmoidin H than to doxorubicin, which was used as a positive control in the study [14]. These results are encouraging for the further evaluation of more potent analogs against multidrug resistant models. 

### 3.2. Pterocarpans

Pterocarpans extracted from the roots of the plant also appeared promising as potential anticancer agents. All three compounds displayed IC_50_ in the micromolar range (from 1.5 to 19 µM; Table 2). Erybraedin A and C are prenylated pterocarpan isomers. Erybraedin A appears to be two to three times more potent than erybraedin C in terms of its IC_50_ (Table 2). In non-small cell lung cancer (NSCLC) cells, Erybraedin A inhibits cell adhesion to fibronectin via the inactivation of the protein tyrosine kinase Src and its interaction with β1 or β3 integrins, leading to cell death by anoïkis [28]. Src is a well-known key oncoprotein involved in cancer cell proliferation and survival through the activation of PI3K/Akt, MAPK, and Stat3 but also in adhesion, migration and angiogenesis by modulating IL-8 and VEGF expression and cytoskeletal formation [83]. Therefore, the inhibition of Src is a currently investigated strategy to combat cancers [84]. The studies conducted with erybraedin C were the former, and mainly showed its ability to kill cancer cells by either necrosis or apoptosis according to the cell model used (necrosis with respect to Jurkat leukemia cells [31] and apoptosis in two colon cancer models [30]). Whether erybraedin C also affects Src function remains to be investigated. Indeed, the modification of the position of the prenylated group on one of the rings may change its reactivity. 

Phaseollin, the third pterocarpan investigated to date, was first described to exert promising in vitro cytotoxic properties, according to an IC_50_ in the low micromolar range and its ability to induce caspase 3- and 7-dependent apoptosis [25]. However, Iranshahi et al. [26] found in 2012 a less potent activity against the prostate cancer cells PC3 (with an IC_50_ higher than 10 µM). This compound structure harbors the same skeleton to that of erybraedin A but the prenylation differs: the prenylation on ring A is cyclized with the hydroxyl group, whereas the prenylation on the phenolic ring 4 is absent.

### 3.3. Tripterpenes

#### 3.3.1. Oleanolic Acid (OA)

OA is a triterpene isolated from *E. senegalensis* by Wandji et al. in 1995 [22]. Its anticancerous potential relies on its cytotoxic effects against various cancer cells by the induction of apoptosis, autophagy, mitophagy and its antimigratory effects as detailed below. The cytotoxic effects of OA have been described in many cancer cell types (Table 2). Accordingly, the following sections may be non-exhaustive.

***OA induces autophagy***. OA activates autophagy, which is characterized by autophagosome formation and the inhibition of mTOR phosphorylation in hepatocellular carcinomas SMMC-7721, gastric cancers SGC-7901 (high grade metastasis), MGC-803 (gastric adenocarnoma) and BGC-823, as well as in T24 urothelial bladder carcinoma [57,65]. This effect occurs through the inhibition of the PI3K/AKT and ERK/p38 MAPK signaling pathways on the one hand, and the activation of the AMPK (AMP activated protein kinase) pathway on the other hand, especially in gastric cancers [65]. In T24 bladder cancer cells, the activation of the AMPK pathway is followed by the stimulation of unc-51 like autophagy-activating kinase 1 (ULK1) [57]. OA has been reported to induce mitophagy in A549 lung cancer cells at 44 µM leading to cell death [85]. Again, in lung cancer cells, OA induces the expression of miR-122 up to 9.9-fold after treatment with 132 µM OA for 8 h [86]. It is well known that MiR-122 induces radiosensitization in non-small cell lung cancer cell lines that are eventually subjected to G0/G1 or G2/M arrest and lose their high proliferation rate [87]. 

***OA induces apoptosis***. OA may also induce apoptosis via the mitochondrial route by altering the potential of the mitochondrial membrane and releasing caspase activators, such as cytochrome c, into the cytoplasm [88]. Intrinsic mitochondrial pathway can be induced by OA even under conditions of hypersalinity (0.26M NaCl), which does not promote cytochrome c release, and which is associated with the overconsumption of glucose and the production of aerobic lactate. AO was shown to induce apoptosis and reverse this metabolic abnormality in high-grade MDA-MB-231 breast cancer cells exposed to hypersalinity [89]. Li et al. 2019 reported the antiproliferative effect of OA on gastric cancer cells MKN-45 and SGC-7901 by blocking aerobic glycolysis following the suppression of key glycolysis enzymes via the inhibition of HIF-1α and YAP (yes-associated protein), an effector protein involved in the proliferation, survival and growth of cells [64]. OA at 219 µM induces the cell death of non-hormone-dependent DU145 prostate cancer cells, as well as hormone-positive MCF-7 metastatic breast cancer (ER^+^/PR^+^) and U87 glioblastoma cells through mitochondrial pathways, with the increased expression of p53, Bax-associated cytochrome c release, caspase-3 activation and PARP cleavage. These effects are mediated through the modulation of the ERK/JNK/AKT pathway [53]. In contrast, in T24 bladder carcinoma cells, OA activates apoptosis via the Akt/mTOR/S6K and ERK1/2 signaling pathways [56]. In HepG2 cells, the inhibition of the JNK/p38 pathway was also involved in OA-induced apoptosis, while sparing normal liver AML12 cells [90]. In these cells, OA may induce both apoptosis and autophagy, supported by an increase in Bax and a decrease in Bcl-2 and by the activation of Akt/mTOR phosphorylation, a decrease of Bcl-2 and an increase of Beclin-1 [61]. The simultaneous induction by AO of apoptosis and autophagy on HCCs has also been demonstrated by Hosny et al. in 2021, using a 7,12-Dimethylbenz-[a]-anthracene induced experimental model [91]. Currently, the inhibition of autophagy by specific inhibitors increases the likelihood of apoptotic death in these cells [61].

***OA induced ferroptosis***. OA activates ferroptosis in Hela cells by promoting ACSL4 expression, thereby reducing the survival rate of these cells [92]. Indeed, acyl-CoA synthetase long-chain family member 4 (ACSL4) is an important contributor to ferroptotic cell death, a recently identified caspase-independent form of regulated cell death that is involved in several physiological and pathological processes. Iron-dependent lipid peroxidation followed by the accumulation of lethal lipid ROS are key features of this newly described cell death type [93].

***OA inhibits migration, invasion and angiogenesis***. The migration and invasion of cancer cells play crucial roles in metastasis. OA, in addition to its cytotoxic effects, is able to inhibit the migration and invasion of hepatocellular and colorectal cancer cells, resulting from the impairment of the epithelial-mesenchymal transition process (EMT) in liver cancer cells HepG2 and PLC-PRF-5 [58,94]. This effect seems to result from the activation of inducible NO synthetase (iNOS) by OA that further leads to increase NO production, and, consequently, the nitrogenation of key proteins involved in the EMT [58]. 

Angiogenesis supports the continued growth of a solid tumor and promotes hematogenous metastasis [95]. OA decreases the invasion, migration, tubing and vascular germination of HUVECs cells, supporting its potential anti-angiogenic effects [63,96]. Angiogenesis is mediated by multiple intracellular signaling pathways, notably, signal transducer and transcription activator 3 (STAT3) and sonic hedgehog transmission cascades (SHH), whose aberrant activation promotes the expression of various critical angiogenic signals, including VEGF-A and b-FGF [97,98]. Thus, inhibition of these pathways by OA suppressed angiogenesis in a HT-29 colorectal carcinoma preclinical model [63]. OA reduced the invasion and migration of both HUVECs and HCT-116 colorectal cancer by blocking phosphorylation or lowering the level of vascular endothelial growth factor receptor-2 (VEGFR2) via the inhibition of MEK/ERK/JNK pathway [96].

#### 3.3.2. Erythrodiol and Maniladiol

Maniladiol and erythrodiol are two other isomeric diol triterpenes isolated for the first time from the stem bark of *E. senegalensis* by Wandji et al. in 1995 [22]. The cytotoxic effects of maniladiol and erythrodiol have been demonstrated on numerous cancer cell lines, with a respective mean IC_50_ of 15 µM and <36 µM (Table 2). Noteworthy, erythrodiol is the alcohol form and the precursor of oleanolic acid in the plant [47]. Except for Kim et al. 2018 [53], who determined a much higher IC_50_ for OA than in the other studies, erythrodiol and OA display very similar in vitro potencies, suggesting that one may also be the precursor of the other in cancer cells. Accordingly, erythrodiol appeared to act also by inducing apoptosis, which has been observed in HT-29 colon carcinoma cells, MDA-MB-231 breast cancer and U937 lymphoma [46,47]. In these two latter models, apoptosis resulted from extensive DNA damage and activation of the ROS/JNK pathway [46]. In addition to the activation of the ROS/JNK pathway, erythrodiol causes disorganization of the actin cytoskeleton in various glioma models that may result in decreased migration [99].

Maniladiol is an isomer of erythrodiol that appears to be more potent, at least on the basis of the few cell lines investigated to date (Table 2). Unfortunately, the mechanism of its cytotoxic activity has not yet been studied in depth, at least to our best knowledge.

## 4. In Vivo Preclinical Studies of *E. senegalensis* Compounds

Of the many secondary metabolites isolated from *E. senegalensis*, only oleanolic acid and alpinumisoflavone have been studied in vivo for their antitumor properties.

### 4.1. In Vivo Studies of Antitumor Activities of Oleanolic Acid (OA) 

OA biological properties have been extensively studied. In addition, OA has been the starting compound for a plethora of derivatization. This section may thus be non-exhaustive and is focused on in vivo data of OA itself. We found around fifteen studies reporting the antitumor effects of OA in preclinical cancer models. OA has been administered generally by the oral or intraperitoneal route, according to its solubility issues for intravenous administration. Doses varied from 12.5 mg/Kg to 150 mg/Kg every day or every two days. OA induced tumor growth retardation in subcutaneous PC3 and DU145 prostate cancer bearing mice [53]. In both studies, the Akt pathway seemed to be involved, as assessed in vitro with respect to the DU145 model, and in vivo with the PC3 model, whose tumors displayed decreased p-Akt levels, as well as decreased Ki67, cyclin D1 and Bcl2 levels [53]. Apoptosis was clearly demonstrated in subcutaneous HepG2 hepatocarcinoma by means of caspase 9 and 3 staining, and in HT-29 colon cancer and A375SM melanoma xenografts by means of TUNEL staining [100,101,102]. In both HepG2 and HT-29 models, ERK/p53/p21 seemed to be a key pathway leading to the altered expression levels of BCL-2 family members [100,102]. 

Significantly reduced tumor growth was, in contrast, attributed to the induction of autophagy in subcutaneous MGC-803 gastric cancer and K-Ras-transformed MCF10A breast xenografts, as demonstrated by a greater accumulation of autophagy-related proteins Beclin-1 and LC3-II within tumors of the OA-treated group of mice [65].

Xiaofei et al. demonstrated that OA inhibits cervical cancer Hela tumor growth through modulation of the ACSL4 ferroptosis signaling pathway, as assessed by the increase in Fe^2+^ and ROS and a decrease in GSH within the treated tumors [92]. 

In addition, treatments with 50 to 150 mg/Kg OA of gallbladder, sarcoma and lung cancer-bearing mice also resulted in decreased tumor volume and/or tumor weight [103,104,105].

The antimetastatic and anti-angiogenic effects of OA have been evaluated in vivo in various mouse tumoral models. In the HT-29 colon cancer subcutaneous xenografts, OA inhibited angiogenesis by suppressing STAT3 and Sonic Hedgehog signaling pathways that control the expression of critical angiogenic stimuli, such as VEGF-A and b-FGF [98]. Similarly, in the HCT-116 model, OA suppressed VEGFR2 phosphorylation, resulting in MEK/ERK/JNK inhibition [96]. The antimetastatic effects of OA were highlighted notably by the study of Wang et al., in which the administration of 50 mg/Kg/day to subcutaneous PLC hepatocarcinoma-bearing mice decreased not only primary subcutaneous tumor size but also the number of lung metastases [58]. Those effects were attributed to the inhibition of the epithelial-mesenchymal transition (EMT), supported by the in vivo higher expression levels of E-cadherin, decreased expression levels of vimentin as well as MMP-2 and -9 [58]. One study also showed a decrease in lung pseudometastases developed after the intravenous injection of melanoma cells, due to an intranasal daily treatment with 5 to 10 mg/Kg of OA [106].

### 4.2. In Vivo Antitumor Studies of Alpinumisoflavone (AIF) 

This chapter summarizes six in vivo studies of AIF antitumor effects conducted on various human subcutaneous xenograft models, including the HCT-116 colon cancer, the KYSE30 and Eca109 esophageal cancer models, the TPC-1 hepatocarcinoma model and the 786-O clear cell renal carcinoma model. AIF was administered at doses ranging from 20 mg/kg/day to 100 mg/kg/day by the intraperitoneal or oral route for a maximum of 30 days of treatment. Importantly, no toxicity in terms of the body weight of the animals nor in terms of the conventional hematoxylin eosin histological evaluation of the organs could be evidenced, suggesting that AIF is well tolerated [36,40,45,73]. AIF treatments induced a decreased tumoral growth in a dose-dependent manner, confirmed by tumor volume measurements and/or tumor weight after resection. In colon [40], esophageal [73] and renal carcinomas [36], this growth impairment was associated wit increased intratumoral apoptosis, as assessed by increased TUNEL staining and cleaved caspase 3 levels. In addition, the Ki67 proliferating marker was also decreased in a dose-dependent manner in the colon cancer model [40] along with decreased Bcl-2 and increased Bax levels.

## 5. Discussion

This study revealed that the identified secondary metabolites of the *E. senegalensis* belonged to six families, i.e., alkaloids, flavonoids, isoflavonoids, pterocarpans, triterpenes and coumarins. Representatives of tannins, emodols and anthracenosides were not found in this review, although such compounds are commonly found in various *E. senegalensis* extracts [107,108]. The most studied parts of the plant as sources for the isolation of substances were the stem bark, roots and seeds, according to the traditional medicinal use of this species: traditional health practitioners rarely use the leaves alone without them being associated with the bark or the roots [8].

### 5.1. Cytotoxic Alkaloids Isolated from E. senegalensis

Erysodine, glucoerysodine and hypaphorine were the only alkaloids isolated from the seeds of *E. senegalensis*. The genus *Erythrina* counts long-known alkaloid-containing plants, e.g., *E. crista galli* [109] and *E. abyssinica* [110]; notably, within their seeds, there are more than in their other parts, such as observed here with respect to *E. senegalensis*. *Erythrina* alkaloids are characterized by a tetracyclic spiroamine system with four linked rings, labeled A, B, C and D, known as the erythrinane skeleton [111]. Herein, we showed that erysodine was weakly active against hepatocarcinoma cells (IC_50_ ranging from 39 µM to 67 µM) and Jurkat cell lines (IC_50_ 39 µM) (Table 2) [48]. Erysodine or erysovine showed an enhanced activity when combined with tumor necrosis factor-related apoptosis-inducing ligand (TRAIL) in Jurkat cells [112] but its mechanism of action has not yet been elucidated. Glucoerysodine exerts antiplasmodial effects [113] while hypaphorin exerts anti-inflammatory [114,115], anti-osteoclastogenic [116], anti-obesity and anti-diabetic effects [117], but their potential activities towards cancer cell lines have not been investigated to date. 

### 5.2. Potential Anticancerous Isoflavonoids and Flavonoids Isolated from E. senegalensis

Isoflavonoids accounted for 50% of all known secondary metabolites of *E. senegalensis*. Isoflavonoids have been shown to display numerous biological properties of therapeutic interest [118,119]. 8-Prenylluteone has anti-HIV properties [120], but its potential anticancerous effects have not yet been studied (Table 1). Erysenegalenseins are isoflavonoids typical of the species *E. senegalensis*. Of these, only erysenegalenseins E and M have been tested and have been shown to exhibit cytotoxic properties. Erysenegalenseins N, O and D are antiviral, notably against HIV [120] but the biological properties of erysenegalenseins B, C, F, G, H, I, J, K and L remain poorly investigated to date. 

Alpinumisoflavone (AIF), derrone, neobavaoflavone, sigmoidin H, warangalone and senegalensin were already previously described as being endowed with in vitro anticancerous properties [14,29,34,41]. Analysis of the IC_50_ shows that the isoflavones AIF, erysenegalensein E, warangalone and derrone are poorly active against the resistant cell model, the KB-3-1 cell line, in comparison to the KB cell line. Warangalone, AIF and derrone are dimethylpyrano-isoflavones. The first two showed, however, better activity than derrone on KB-3-1 cells (Table 2). They differ from derrone by the position of dimethylpyrano on ring A (position six to seven for the two first compounds, and position seven to eight for derrone). Apart from any structure–activity relationship study, this observation could suggest the non-negligible role of the “dimethylpyrano” position on the A ring. In addition, the prenylation in C-8 of the A ring (warangalone) did not seem to reduce the compound’s activity more than the oxidation of the prenyl group at the same position (erysenegalensein E).

Derrone and AIF display moderate to low activity on hormone-dependent breast cancer cell lines (MCF-7, T47D) [50,121] but appear more potent on non-hormone-dependent breast cancers (MDA-MB-231) [38], while sigmoidin H and neobavaisoflavone were inactive on these models [14]. Structurally, AIF and derrone are close to genistein. Genistein and its derivatives behave as phytoestrogens, inducing cell proliferation at low doses (<5 µM) and cell death at high doses (>10 µM) in hormone-dependent (ER+) breast cancer cells [122]. Such phytoestrogenic properties may explain their lower activity towards hormone-dependent models in comparison to non-hormone-dependent models [123]. Accordingly, AIF and neobavaisoflavone have been endowed with estrogenic activity [124,125]. The variation between the responses of the cellular models to each of those compounds may depend on the expression levels of the different estrogen receptor-subtypes in each cell line, and the affinity and selectivity of the compounds for each of them [125]. Regarding prostate cancers, AIF and derrone were reversely inactive on hormone (androgen)-independent PC-3 cells [26,49,126] but their activity towards hormone-dependent prostate cancer models has not been tested yet. On the other hand, their analogs that are prenylated in C-8 of cycle A, such as warangalone (IC_50_: 7 µM) and erysenegalensein M (IC_50_: 8 µM), induced powerful antiproliferative effects on LNCaP cells (androgen-sensitive) [29]. 

Neobavaisoflavone and sigmoidin H are the only isoflavones from *E. senegalensis* that are prenylated in position 2’ of cycle B described to date. The prenyl group and the dimethylpyrano are attached in position 2’, respectively, for neobavaisoflavone and sigmoidin H (Figure 1). The position of such groups on the B ring and not on the A ring appears to reduce the chemoresistance of U87MG.ΔEGFR glioblastomas. Tchokouaha et al. 2010 revealed that number and binding site of prenyl groups influenced the biological activity of flavonoids [29]. In this case, the binding of dimethylpyrano and prenyl groups on cycle A (AIF, derrone) would enhance cytotoxic activity, rather than on cycle B (neobavaoflavone, sigmoidin H), which would nevertheless provide an advantage against EGFR-mutated GBM.

### 5.3. Potential Anticancerous Pterocarpans Isolated from E. senegalensis

Erybraedins A and C are isomers differing only in the position of the two prenyl groups on the different rings, while phaseollin is a dimethylpyrano-pterocapane. Erybraedin A was more active than Erybraedin C on many cancer cell lines including lung cancer, cervical cancer and breast cancer. The excellent biological properties of erybraedins A and C are thought to be explained by their prenylation in the C-4 position, which is shared by the two compounds. According to authors [27,127], the C-4 or C-2-prenylation of pterocarpans enhances their biological activities, but the current results suggest that erybraedin C, bearing these two prenylations, is less potent than erybraedin A (Table 2; mean IC_50_ of 17 µM in comparison to 8 µM, respectively). One of the two prenyl groups of erybraedin C is essential in inhibiting human topoisomerase I [128]. The attachment of a dimethylpyrano group to C-4 in the case of phaseollin also appears to enhance cytotoxic activity on non-hormone-dependent PC-3 prostate cancer (IC_50_ 9 µM) [26] and on hepatocellular carcinoma models (IC_50_ 1.5 µM) in comparison to erybraedins. According to its potency, phaseollin merits further investigations. 

### 5.4. Cytotoxic Pentacyclic Triterpenes Isolated from E. senegalensis

The cytotoxic triterpenes isolated from *E. senegalensis* to date are oleanolic acid (OA) and the two triterpenes diol maniladiol and erythrodiol. Overall, OA is the most studied one, both in vitro and in vivo, on various cancer models, including cancers for which the therapeutic arsenal was currently scarce, despite its high IC_50_ values (mean IC_50_ of 73 µM). This may be due to its widespread presence in numerous plants and food products and the very wide range of potential benefits on health. More importantly, OA appeared to be non-toxic in mice at doses associated with significant antitumor effects in numerous models. It thus appears still as an interesting candidate, notably when considering its various cytotoxic mechanisms of action described above and which differ from those of the old drugs still currently used in modern therapy. 

Diol triterpenes (maniladiol and erythrodiol) appeared to display better activity than oleanolic acid (the oxidized form of erythrodiol), based on their in vitro potency, according to their IC_50_ concentrations. Maniladiol is a pentacyclic diol triterpene which differs from erythrodiol in the positions of both its hydroxyl and both dimethyl groups. However, their in vivo efficiencies has not been studied yet and would merit further investigations. 

### 5.5. Current Status, Limitations and Perspectives

According to this systematic review, several main findings and limitations merit to be discussed. Indeed, among the 42 metabolites identified in *E. senegalensis*, only erysenegalenseins have been identified specifically in this species, at least to date. However, the few results available on their potential anticancerous activities (i.e., an IC_50_ of 8 µM for erysenegalensein M) suggest that they might display better anti-proliferative effects than numerous other isoflavonoids. Further studies on the other erysenegalenseins, as well as mechanistic studies, are thus needed to better identify the specific potential of these secondary metabolites to combat cancers. The fact that for now they have been isolated solely in *E. senegalensis* and the relatively low yield of the current extraction methods (from 0.12 mg/Kg of dried plant material to 67 mg/Kg [10,11,16,17,18,22]) may impede their evaluation as potential anticancer agents.

Although not specific to *E. senegalensis*, this species appeared very rich in numerous other flavonoids and isoflanoids, supporting the use of this plant in traditional practice. In particular, AIF has shown promising results in preclinical models. AIF can be easily synthesized with good yield [129] to supply samples for further biological screening [130]. Other flavoinoids have been tested alone or in combination to combat colon cancer and their use may actually be envisaged in chemoprevention, therapeutic purposes and as palliative care to avoid side effects of chemotherapy [131].

Regarding OA, the other metabolite found notably in *E. senegalensis* and having been evaluated in preclinical models, its main issues relate to its poor water solubility and its significant efflux that have limited its widespread clinical use. Three strategies are used to date to overcome those drawbacks: (i) formulations, (ii) derivatization and (iii) drug combinations. Plenty of papers may be found supporting the considerable interest in this compound or its derivatives. Indeed, formulations of OA in nanoparticles of various compositions have improved its pharmacokinetics [132] and its efficacy on various cancer models, including hepatocellular carcinomas [94,133,134], breast cancers [135], skin cancers [136,137] and lung cancers [138]. Formulation also allowed for a more targeted release of OA [139] and development of combinations between OA and marketed anticancer drugs such as paclitaxel [140] and doxorubicin [134], but also with other potential therapies such as sodium heparin [94] and other cytotoxic triterpenes like ursolic acid [141]. Despite their better in vitro potency, the effects of maniladiol and erythrodiol against cancer models have been poorly investigated. Interest seems to be turned onto some less hydrophobic derivatives [142,143,144]. Noteworthy, the introduction of an oxygen substituent into the C-ring increases the biological activities, including cytotoxic effects [145]. 

Finally, it is important to note that very few studies have been conducted to identify other chemical family secondary metabolites from *E. senegalensis*. We are currently characterizing the composition of several extracts of *E. senegalensis,* harboring interest in their invitro activities against cancer cell models through conventional reaction characterization and LC-MS. We thereby identified glycosylated and aglycone triterpenes as well as coumarinic compounds (unpublished data, work in progress). Those later might be of high interest according to the the last review of Akkol et al. in 2020 [146].

## 6. Materials and Methods

Between October 2020 and February 2021, scientific databases such as PubMed and Google Scholar were consulted using the terms “*Erythrina senegalensis*” or “*Erythrina senegalensis* AND cytotoxicity” or “*Erythrina senegalensis* AND cancer” as descriptors after verification via “MeSH”. All indexed scientific articles have been actively researched and exploited. All scientific articles, in where at least one chemical substance had been isolated and identified from the plant *E. senegalensis,* were retained.

The exact UIPAC name of each secondary metabolite isolated from *E. senegalensis,* as well as its chemical formula, were obtained from the PubChem database. From then on, the chemical structures were redrawn using ChemDraw Pro 16.0 software. The exact denomination of each secondary metabolite was used in turn as a keyword for an active search for scientific articles related to the anticancer activities established through Pubmed and Google scholar. All the articles dealing with the evaluation of the anticancer activity of a substance isolated from *E. senegalensis* were retained and used even when the source of the compound differed.

## 7. Conclusions

Our study revealed that 42 secondary metabolites have been isolated from *E. senegalensis* to date. There are 19 of which that exhibit potentially anticancerous effects, belonging to alkaloids, triterpenes and prenylated isoflavonoids, flavonoids and pterocarpans. 

Although only erysenegalenseins appear to be specific to this species, the peculiarity of many of its secondary metabolites reside in the molecular mechanism of action. They are able to induce non-apoptotic cell death, including autophagy, mitophagy and pyroptosis, which differs far from those of the old pro-apoptotic chemotherapeutic drugs. 

## Figures and Tables

**Table 1:** Isolated compounds from *E. senegalensis* described to date with their origin, obtention method and whether their potential anticancerous activities were evaluated; YES: anticancerous activity demonstrated, at least in vitro, NO: tested but no in vitro anticancerous activity observed; NT: not tested.

Compounds	Formula	Chemical Family	Part of the Plant	Solvent Used	Ref.	Anticancerous Activity
Erysodine	C_18_H_21_NO_3_	alkaloid	Seed	MeOH re-extracted by CH_2_Cl_2_	[10]	YES
Hypaphorine	C_14_H_18_N_2_	alkaloid	Seed	MeOH re-extracted by CH_2_Cl_2_	[10]	NT
Glucoérysodine	C_24_H_31_NO_8_	Alkaloid	Seed	MeOH re-extracted by CH_2_Cl_2_	[10]	NT
Erysenegalensein F	C_25_H_24_O_7_	Epoxy isoflavone	Stem bark	MeOH re-extracted by CH_2_Cl_2_	[11]	NT
Erysenegalensein G	C_25_H_24_O_7_	Epoxy isoflavone	Stem bark	MeOH re-extracted by CH_2_Cl_2_	[11]	NT
Erythrinasinate	C_38_H_66_O_4_	Cinnamate	Stem bark	n-hexane re-extracted by CHCl_3_	[12]	NO
Carpachromene	C_20_H_16_0_5_	Flavone	Root	CH_2_Cl_2_	[13]	YES
Sigmoidin H	C_21_H_20_O_5_	Isoflavanone	Stem bark	MeOH/CH_2_Cl_2_ (1:1)	[14]	YES
Erysenegalensein B	C_25_H_28_O_7_	Isoflavanone	Stem bark	MeOH re-extracted by CH_2_Cl_2_	[15]	NT
Erysenegalensein C	C_25_H_28_O_7_	Isoflavanone	Stem bark	MeOH re-extracted by CH_2_Cl_2_	[15]	NT
Alpinumisoflavone	C_20_H_16_O_5_	Isoflavone	Stem bark	MeOH re-extracted by CH_2_Cl_2_	[16]	YES
Derrone	C_20_H_16_O_5_	Isoflavone	Stem bark	MeOH re-extracted by CH_2_Cl_2_	[16]	YES
8-prenylluteone	C_25_H_26_O_6_	Isoflavone	Stem bark	MeOH re-extracted by CH_2_Cl_2_	[17]	NT
Erysenegalensein D	C_25_H_26_O_7_	Isoflavone	Stem bark	MeOH re-extracted by CH_2_Cl_2_	[17]	NT
Erysenegalensein E	C_25_H_26_O_6_	Isoflavone	Stem bark	MeOH re-extracted by CH_2_Cl_2_	[17]	YES
Erysenegalensein H	C_25_H_26_O_7_	Isoflavone	Stem bark	MeOH re-extracted by CH_2_Cl_2_	[18]	NT
Erysenegalensein I	C_25_H_26_O_7_	Isoflavone	Stem bark	MeOH re-extracted by CH_2_Cl_2_	[18]	NT
Erysenegalensein M	C_25_H_24_O_7_	Isoflavone	Stem bark	MeOH re-extracted by CH_2_Cl_2_	[10]	YES
Erysenegalensein L	C_25_H_24_O_7_	Isoflavone	Stem bark	MeOH re-extracted by CH_2_Cl_2_	[10]	NT
Lupinifolin	C_25_H_26_O_5_	Isoflavone	Stem bark	MeOH re-extracted by CH_2_Cl_2_	[18]	YES
Neobavaisoflavone	C_20_H_18_O_4_	Isoflavone	Stem bark	MeOH/CH_2_Cl_2_ (1:1)	[14]	YES
6,8-diprenylgenistein	C_25_H_26_O_5_	isoflavanone	Stem bark	n-hexane re-extracted by CHCl_3_	[19]	NT
2,3-dihydro-auriculatin	C_25_H_26_O_6_	isoflavanone	Stem bark	n-hexane re-extracted by CHCl_3_	[19]	NT
Auriculatin	C_25_H_24_O_6_	isoflavanone	Stem bark	n-hexane re-extracted by CHCl_3_	[19]	NT
Erythrisenegalone	C_25_H_26_O_5_	Flavanone	Stem bark	n-hexane re-extracted by CHCl_3_	[20]	YES
Senegalensein (Lonchocarpol A)	C_25_H_28_O_5_	Flavanone	Stem bark	n-hexane re-extracted by CHCl_3_	[21]	YES
Warangalone (Scandenone)	C_25_H_24_O_5_	isoflavanone	Stem bark	n-hexane re-extracted by CHCl_3_	[20]	YES
Erysenegalensein J	C_25_H_24_O_7_	Isoflavanone	Stem bark	MeOH re-extracted by CH_2_Cl_2_	[22]	NT
Erysenegalensein K	C_22_H_18_O_6_	Isoflavanone	Stem bark	MeOH re-extracted by CH_2_Cl_2_	[22]	NT
Erysenegalensein N	C_25_H_26_O_7_	Isoflavone	Stem bark	MeOH re-extracted by CH_2_Cl_2_	[16]	NT
Erysenegalensein O	C_25_H_26_O_7_	Isoflavone	Stem bark	MeOH re-extracted by CH_2_Cl_2_	[16]	NT
Senegalensin	C_25_H_26_O_6_	Isoflavone	Stem bark	n-hexane re-extracted by CHCl_3_	[23]	YES
Erybraedin A	C_21_H_20_O_5_	Pterocarpan	Root	CH_2_Cl_2_	[13]	YES
Erybraedin C	C_21_H_20_O_5_	Pterocarpan	Root	CH_2_Cl_2_	[13]	YES
Eryvarine K	C_21_H_22_O_5_	Pterocarpan	Root	CH_2_Cl_2_	[13]	NT
Phaseollin	C_20_H_18_O_4_	Pterocarpan	Root	CH_2_Cl_2_	[13]	YES
Shinpterocarpin	C_20_H_18_O_4_	Pterocarpan	Root	CH_2_Cl_2_	[13]	NT
Erybraedin D	C_25_H_26_O_4_	Pterocarpan	Root	CH_2_Cl_2_	[13]	NT
Cornulacic acid	C_30_H_48_O_3_	Triterpene	Stem bark	MeOH re-extracted by CH_2_Cl_2_	[22]	NT
Erythrodiol	C_30_H_50_O_2_	Triterpene	Stem bark	MeOH re-extracted by CH_2_Cl_2_	[22]	YES
Maniladiol	C_30_H_50_O_2_	Triterpene	Stem bark	MeOH re-extracted by CH_2_Cl_2_	[22]	YES
Oleanolic acid	C_30_H_48_O_3_	Triterpene	Stem bark	MeOH re-extracted by CH_2_Cl_2_	[22]	YES

**Table 2:** In vitro cytotoxic activity of secondary metabolites isolated from *E. senegalensis.* *: mouse cell line; ** rat cell line.

Compound	Cancer Type	Cell Line	Specificity of the Cell Line	Time of Treatment	IC_50_ (µM)	Ref.
Carpachromene	Lymphoma	Raji		NS	0.029	[24]
	Liver cancer	HepG2		NS	0.024	[24]
	Liver cancer	PLC/PRF/5		NS	0.016	[24]
Phaseolin	Rat hepatoma cancer	H411E **		24 h	1.5	[25]
	Prostate cancer	PC3		24 h	10	[26]
Erybraedin A	Breast cancer	BC		NS	7	[27]
	Lung cancer	A549		24 h	1–5	[28]
		H1299		24 h	1–5	[28]
		H226B		24 h	1–5	[28]
		NCI-H187		24 h	5	[27]
	Breast cancer	BC		24 h	7	[27]
	Cervix carcinoma	KB		NS	13	[27]
Erysenegalensein M	Breast cancer	MCF-7		NS	8	[29]
	Prostate cancer	LNCaP		NS	8	[29]
Erybraedin C	Colon cancer	HT29	MMR +/+ p53 −/− Bcl-2 +/+	NS	5	[30]
		LoVo	MMR −/− p53 +/+ Bcl-2 −/−	NS	4	[30]
	Leukemia	Mono-Mac-6		72 h	29	[31]
		CD4+ Jurkat T *	Bcl-2 overexpression	72 h	18	[31]
		CD4+ Jurkat T		72 h	21	[31]
	Cervix carcinoma	KB		72 h	24	[31]
Maniladiol	Renal cancer	RFX393		NS	7	[32]
	Lung cancer	Lu1		NS	31	[33]
	Breast cancer	MCF-7		NS	31	[33]
		T-47D		NS	10	[32]
	Prostate cancer	LNCaP		NS	26	[33]
Warangalone	Breast cancer	MCF-7		NS	7	[29]
	Cervix carcinoma	K-B-3-1		NS	73	[34]
	Endometrial cancer	Ishikawa		NS	7	[29]
	Leukemia	HL-60		72 h	30	[35]
	Prostate cancer	LNCaP		NS	7	[29]
Alpinumisoflavone	Kidney cancer	RCC4		NS	5–10	[36]
	Carcinoma	KB		72	12	[37]
	Cervix carcinoma	KB-3-1		NS	71	[34]
	Breast cancer	MDA-MB-231		NS	5	[38]
		MDA-MB-231-BCRP	BCRP resistant	NS	66	[38]
		MDA-MB-231-pcDNA		NS	43	[38]
		T47D		NS	5	[39]
	Colon cancer	HCT 116	p53 −/−	NS	36	[38]
		HCT 116	p53 +/+	NS	42	[38]
		SW 480		24 h	5–10	[40]
		SW 480		48 h	5–10	[40]
	Glioma	U87MG		NS	47	[38]
		U87MG.ΔEGFR	Multiresistant	NS	42	[38]
	Liver carcinoma	Bel 7402		48 h	27	[41]
		HepG2		48 h	23	[41]
		Huh7		48 h	14	[41]
		SMMC 7721		48 h	18	[41]
	Leukemia	CCRF-CEM		NS	10	[38]
		CEM/ADR5000	Pgp overexpression	NS	6	[38]
		HL-60		48 h	19	[42]
		K-562		48 h	34	[42]
		MOLT-4		48 h	41	[42]
		P-388 *		NS	13	[43]
	Lung cancer	H1299		24 h	39	[43]
		H2108		24 h	34	[44]
		MRC-5		24 h	53	[44]
Alpinumisoflavone	Esophagal cancer	Eca 109		72 h	10–20	[45]
		KYSE30		72 h	10–20	[45]
Erythrodiol	Breast cancer	MCF-7		120 h	12.5–25	[46]
	Colon cancer	HT29		NS	49	[47]
	Liver carcinoma	HepG2		NS	11	[48]
Erysodine	Liver cancer	HEP-2		NS	66	[48]
	Liver cancer	HepG2		NS	39	[48]
Erysenegalensein E	Cervix carcinoma	KB-3-1		NS	99	[34]
	Cervix carcinoma	KB		NS	15	[37]
Derrone	Colon cancer	HCT 116		24 h	42	[49]
	Prostate cancer	PC3		24 h	45	[49]
	Breast cancer	KPL4		24 h	46	[50]
		MCF-7		24 h	24	[50]
	Cervix carcinoma	HeLa		24 h	31	[50]
		KB-3-1		NS	230	[34]
	Lung cancer	A549		24 h	43	[49]
		H1299		24 h	24	[50]
		H292		24 h	39	[49]
Erythrinasinate	Leukemia	CCRF-CEM		72 h	>70	[51]
		CEM/ADR5000		72 h	>70	[51]
Neobavalisoflavone	Glioma	U87MG.ΔEGFR	Multiresistant GBM	NS	78	[14]
	Leukemia	CCRF-CEM		NS	51	[14]
		CEM/ADR5000	Multiresistant leukemia	NS	43	[14]
	Liver carcinoma	HepG2		NS	110	[14]
Oleanolic acid	Breast cancer	MCF-7/wt	BCR1 expression	72 h	28	[52]
		MCF-7/ADR	BCR1 expression	72 h	44	[52]
		MCF-7		24 h	290	[53]
	Glioma	U87MG		24 h	358	[53]
	Prostate cancer	PC3		72 h	40	[54]
		DU145		72 h	30	[54]
		LNCaP		72 h	25	[54]
		DU145		24 h	246	[53]
	Thyroid cancer	SW 579		NS	42	[55]
	Gall bladder cancer	GBC-SD		72 h	48	[56]
		NOZ		72 h	60	[56]
	Pancreas cancer	Panc-28		24 h	102	[57]
	Bladder cancer	T24		48 h	50	[58]
		EJ		24 h	10–20	[58]
		T24		24 h	8–16	[59]
	Liver cancer	HepG2		NS	30	[60]
		SMC7721		48 h	25	[61]
		HepG2		48 h	21	[61]
		SMMC-7721		24 h	30–60	[62]
	Colon cancer	HCT 116		48 h	88	[63]
		HT29		24 h	25	[64]
	Gastric cancer	MKN-45		24 h	20–30	[65]
		BGC-823		72 h	22	[66]
		MGC-803		72 h	20	[66]
		SGC-7901		72 h	21	[66]
	Leukemia	HL-60		72 h	55	[67]
		HL-60		72 h	9	[68]
	Osteosarcoma	MG63		72 h	100	[69]
		Saos-2		72 h	50	[69]
		MG63		72 h	75	[70]
		Saos-2		72 h	60	[70]
Sigmoidin H	Glioma	U87MG		NS	26	[14]
	Leukemia	CCRF-CEM		NS	98	[14]
		CEM/ADR5000	Multiresistant	NS	100	[14]

## Data Availability

Not applicable.

## Supplementary Materials

The following are available online at https://www.mdpi.com/article/10.3390/plants11010019/s1, Figure S1: Chemical structures of all secondary metabolites isolated from *E. senegalensis* grouped into families.
