# Supplementary material for: Systematic Review of Potential Anticancerous Activities of Erythrina senegalensis DC (Fabaceae)
Source: Plants (Basel). 2021 Dec 22;11(1):19. doi: 10.3390/plants11010019 (PMC8747466; doi:10.3390/plants11010019)
Supplement: Supplementary file 1 [file plants-11-00019-s001.zip › plants-1487372-supplementary.pdf]

A. Isoflavonoids

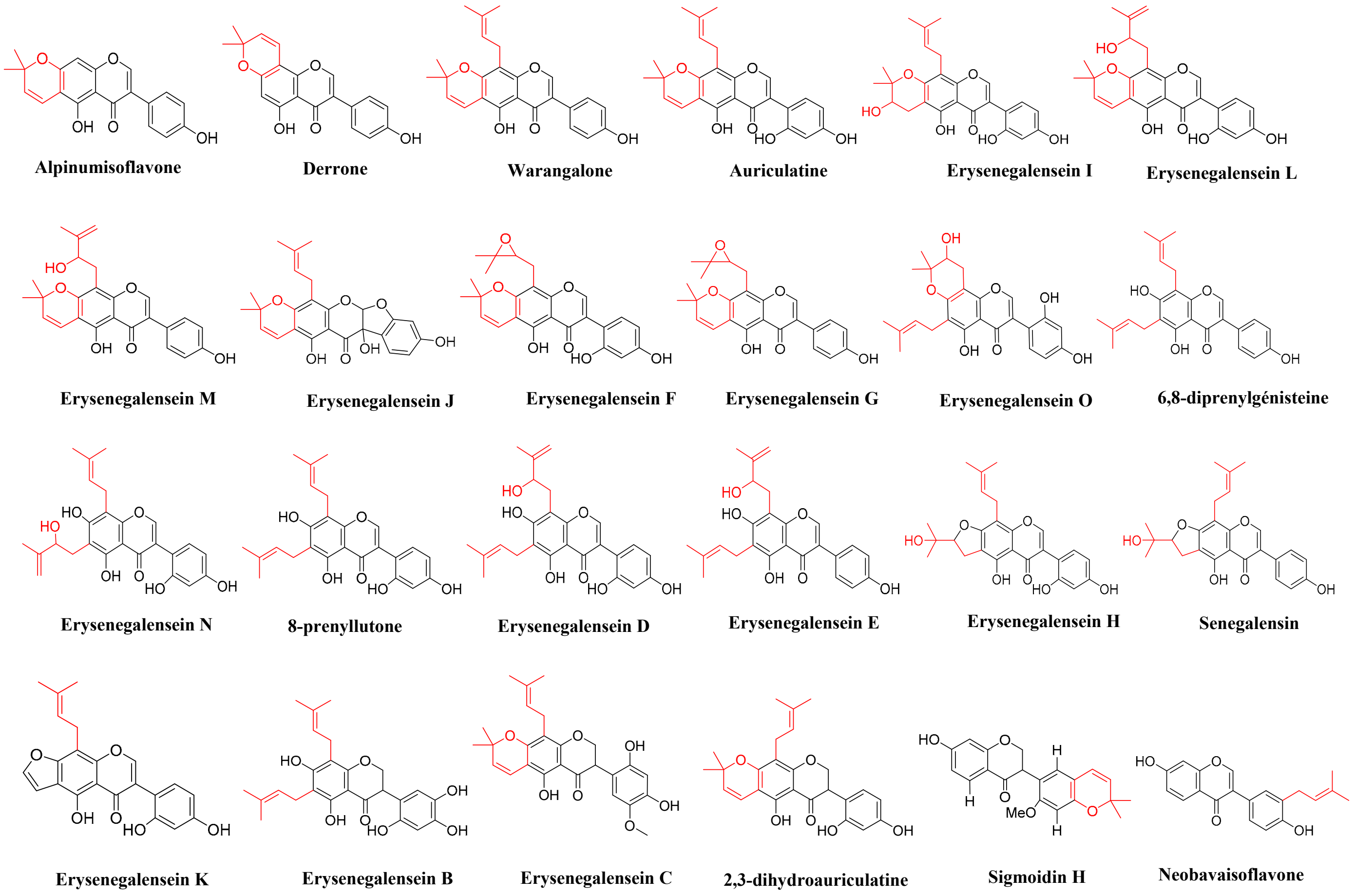

B. Flavonoids

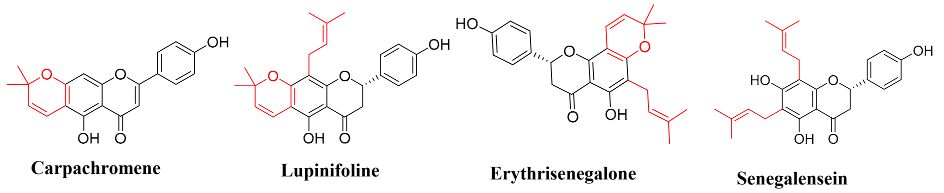

C. Cinnamate

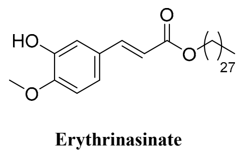

D. Pterocarpans

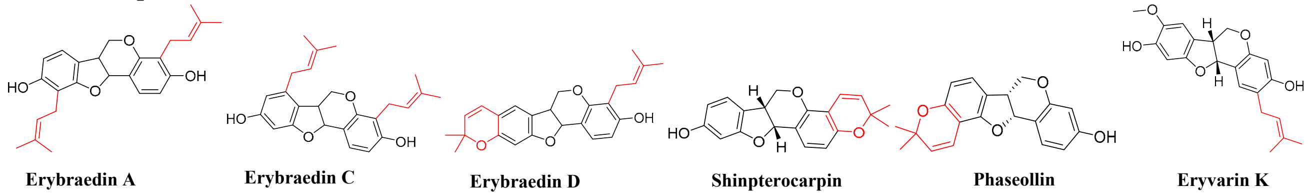

E. Triterpens

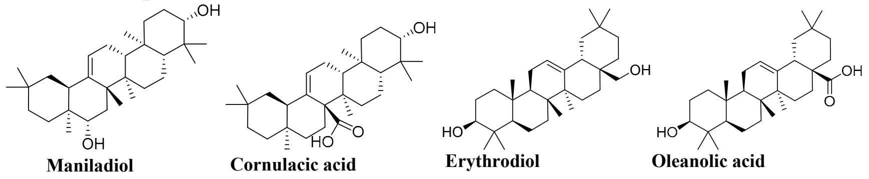

F. Alkaloids

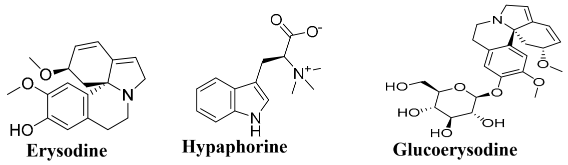

Figure S1: Chemical structures of all secondary metabolites isolated from E. senegalensis grouped into families. The patterns in red indicate the prenylation of the molecule.
